# Extraretinal Spike Normalization in Retinal Ganglion Cell Axons

**DOI:** 10.1523/ENEURO.0504-19.2020

**Published:** 2020-03-19

**Authors:** Alex Fogli Iseppe, Genki Ogata, Jeffrey S. Johnson, Gloria J. Partida, Nicholas Johnson, Christopher L. Passaglia, Andrew T. Ishida

**Affiliations:** 1 Department of Neurobiology, Physiology and Behavior, University of California, Davis, California 95616; 2 Department of Molecular Pharmacology and Physiology, University of South Florida, Tampa, Florida 33620; 3 Department of Chemical and Biomedical Engineering, University of South Florida, Tampa, Florida 33612; 4 Department of Ophthalmology and Vision Science, University of California, Sacramento, California 95817

**Keywords:** axonal spikes, cell type-specific differences, normalization of duration, optic chiasm, optic tract, retina

## Abstract

Spike conduction velocity characteristically differs between myelinated and unmyelinated axons. Here we test whether spikes of myelinated and unmyelinated paths differ in other respects by measuring rat retinal ganglion cell (RGC) spike duration in the intraretinal, unmyelinated nerve fiber layer and the extraretinal, myelinated optic nerve and optic chiasm. We find that rapid spike firing and illumination broaden spikes in intraretinal axons but not in extraretinal axons. RGC axons thus initiate spikes intraretinally and normalize spike duration extraretinally. Additionally, we analyze spikes that were recorded in a previous study of rhesus macaque retinogeniculate transmission and find that rapid spike firing does not broaden spikes in optic tract. The spike normalization we find reduces the number of spike properties that can change during RGC light responses. However, this is not because identical spikes fire in all axons. Instead, our recordings show that different subtypes of RGC generate axonal spikes of different durations and that the differences resemble spike duration increases that alter neurotransmitter release from other neurons. Moreover, previous studies have shown that RGC spikes of shorter duration can fire at higher maximum frequencies. These properties should facilitate signal transfer by different mechanisms at RGC synapses onto subcortical target neurons.

## Significance Statement

Myelination increases spike conduction velocity in axons. To test whether unmyelinated and myelinated segments of axons differ only in propagation speed, we compared spikes in unmyelinated intraretinal and myelinated extraretinal portions of retinal ganglion cell (RGC) axons. Short interspike intervals and illumination increased spike duration intraretinally but not extraretinally. Consequently, light can change only whether and how often extraretinal spikes fire. Notably, this does not eliminate axonal spike duration differences between subtypes of RGC. This would allow previously described properties of narrower and broader spikes to facilitate signal transfer by different mechanisms at RGC synapses onto subcortical target neurons.

## Introduction

Axons are generally described as either myelinated or unmyelinated. Retinal ganglion cells (RGCs) differ markedly from this dichotomy in that their axons are formed by long unmyelinated proximal portions and, in nearly all species examined to date, myelinated distal portions (Bruesch and Arey, 1942; Hildebrand et al., 1985; Stone et al., 1995). The proximal portions are intraretinal, arise from RGC somata or primary dendrites, join fascicles in the innermost sublayer of the retina (the nerve fiber layer), converge at the optic disk, and range in length up to 3.5 mm in rats and ∼20 mm in humans (Ramón y Cajal, 1972; Fukuda, 1977; Curcio and Allen, 1990). The distal extraretinal portions exit the eye, project to numerous subcortical brain areas via the optic nerve, optic chiasm, and optic tract (Berson, 2008; Morin and Studholme, 2014), and are ∼20 mm long in rats and 100 mm long in humans (Sumitomo et al., 1969; Matheson, 1970; Lang and Reiter, 1985; Bernstein et al., 2016).

Consistent with the ability of myelinated axons to conduct spikes faster than unmyelinated axons, the conduction velocities of extraretinal RGC axons are significantly faster than those of intraretinal RGC axons (Granit, 1955; Stone and Freeman, 1971; Stanford, 1987). However, it is not known whether spikes of intraretinal and extraretinal axon segments differ only in conduction velocity. This is of interest because intraretinally recorded RGC spikes change in shape during repeated spike firing (O’Brien et al., 2002; Weick and Demb, 2011) and changes in spike shape alter synaptic efficacy in various preparations (Kusano et al., 1967; Sabatini and Regehr, 1997; Geiger and Jonas, 2000). Moreover, RGCs spike over a broad range of frequencies during changes in illumination (Sakmann and Creutzfeldt, 1969), and increases in instantaneous spike frequency increase the efficacy of signal transfer from at least some RGCs to thalamic neurons (Singer et al., 1972; Mastronarde, 1987; Usrey et al., 1998; Sincich et al., 2007; Weyand, 2007).

Here we examine spikes recorded from the nerve fiber layer, optic nerve, optic chiasm, and optic tract, and test whether the spikes of intraretinal and extraretinal RGC axons differ in three respects known from previous somatic and optic nerve recordings (Hartline, 1938; Cleland and Levick, 1974; Fukuda and Stone, 1974; Hidaka and Ishida, 1998; O’Brien et al., 2002; Kim and Rieke, 2003; Weick and Demb, 2011). Specifically, we test whether spike duration varies with interspike interval, whether the onset of light and/or dark alters spike duration, and whether the axons of subtypes of RGCs generate spikes of different duration. We find that short interspike intervals and the onset of illumination increase spike duration in intraretinal axons but not in extraretinal axons. These results identify a previously unrecognized function of the extraretinal portion of RGC axons, namely to limit the effect of light to altering whether and how often spikes fire extraretinally. In addition, we find that differences among cell types in spike duration of intraretinal axons persist in extraretinal axons. These differences show that the loss of activity- and light-induced spike broadening in extraretinal axons does not result from the generation of identical spikes in the myelinated portion of all RGC axons. This identifies a second function of the extraretinal portion of RGC axons, namely to help establish spike duration phenotypes of different cell subtypes. Notably, the spike duration differences we find between the extraretinal axons of different RGCs resemble spike duration changes that alter the amount of neurotransmitter released per spike in other systems (Sabatini and Regehr, 1997; Geiger and Jonas, 2000).

## Materials and Methods

The experiments reported here were performed on *ex vivo* retinae, *in vivo* optic nerve, *in vivo* optic chiasm, and *in vivo* optic tract.

The *ex vivo* retina recordings were performed at the University of California, Davis. All animal procedures were performed using methods approved by the University of California, Davis, Animal Use and Care Administrative Advisory Committee in accordance with National Institutes of Health guidelines. Long–Evans rats of both sexes [*n* = 45; postnatal day 60 (P60) to P120; weight, 150–250 g; Envigo Bioproducts] were killed by a lethal dose of sodium pentobarbital (75 mg/kg, i.p.; Western Medical Supply). After enucleating and hemisecting the eyes, the retinae were isolated and placed in recording chambers (Partida et al., 2018). We found no consistent difference in RGC spike properties between male and female rats, and therefore pooled the results we obtained from them.

The optic nerve and optic chiasm spikes were recorded *in vivo* at the University of South Florida, using methods approved by the Institutional Animal Care and Use Committee of the University of South Florida in accordance with National Institutes of Health guidelines. Brown Norway rats (*n* = 25; male; weight, 300–400 g; Envigo Bioproducts) were anesthetized by intraperitoneal injection of ketamine hydrochloride (75 mg/kg) and xylazine (7.5 mg/kg), rested on a heating pad (37°C), and maintained under anesthesia for the remainder of the experiment via intravenous infusion of ketamine (30 mg/kg/h), xylazine (1.5 mg/kg/h), and 0.9% physiological saline. Eye movements were blocked by adding gallamine triethiodide (40 mg/kg/h) to the infusate. A tracheotomy was performed to provide mechanical ventilation during paralysis. Body temperature and heart rate were monitored with a rectal thermometer and EKG electrodes, respectively, and data were collected while vital signs remained at normal levels. Animals were killed with Euthasol (50 mg/kg; Virbac AH) at the end of experiments.

The optic tract spikes were recorded *in vivo* at the University of California, San Francisco, using methods approved by the University of California, San Francisco, Institutional Animal Care and Use Committee in accordance with National Institutes of Health guidelines. These spikes were recorded in rhesus monkeys (*n* = 2; unknown sex) during a previously published study of retinogeniculate signal transmission (Sincich et al., 2007). None of the observations presented here duplicate previously published analyses, figures, or graphs.

### Intracellular recording

Spikes that were recorded intracellularly from dissociated RGC somata during a previously published study (Hayashida et al., 2009) are shown to help explain how spike duration was measured. As described in detail in that study, these spikes were elicited and measured in perforated-patch whole-cell current-clamp mode with a discontinuous single-electrode voltage-clamp amplifier (SEC-05LX, npi electronic). The conclusions of the present study are drawn entirely from the extracellularly recorded spikes described below, and none of the intracellularly recorded membrane voltage (*V*_m_) traces and measurements shown here duplicate any previously published analyses, figures, or graphs.

### Multielectrode array recording and spike identification

Spikes were recorded from isolated retinae by use of multielectrode arrays (MEAs) and classified as somatic or axonal based on the shape of their mean waveforms (Terzuolo and Araki, 1961; Meister et al., 1994). Each retina was placed ganglion cell layer down on an MEA (catalog #60MEA200/30iR-ITO, MultiChannel Systems), held in place with a slice harp (catalog #SHD-41/10, Warner Instruments), and superfused at 35–37°C with buffered Ames medium (A1372-25, United States Biologicals). The individual electrodes were 30 μm in diameter and arranged in an 8 × 8 rectilinear grid with 200 μm interelectrode spacing. Analog data were acquired simultaneously from each of the recording electrodes for 60–180 min, bandpass filtered (1 Hz to 3 kHz, 60 dB/decade), and sampled at a rate of 25 kHz/channel. Data that will be termed “spontaneous spikes” were collected in the absence of light stimuli, under steady ambient illumination of 150 lux. “Electrically stimulated spikes” were activated by biphasic current pulses delivered via one of the array electrodes (Sekirnjak et al., 2006). These spikes propagated as rapidly as in other RGCs (Zeck et al., 2011; Greschner et al., 2014; Li et al., 2015). Moreover, we excluded spikes firing at electrodes surrounding single stimulating electrodes on multiple sides (Greschner et al., 2014). The electrically stimulated spikes reported here are likely to have been recorded distal to the axon initial segments (AISs) of RGC axons because the stimulating and recording electrodes were separated by at least 400 μm (i.e., by distances greater than those between RGC somata and AISs; Boiko et al., 2003). Because we could record these spikes in a low Ca^2+^/high Mg^2+^ superfusate (see below) that blocked light-evoked responses, these spikes are likely to have been elicited by direct stimulation rather than by presynaptic inputs (Masland and Ames, 1976). “Light responses” were elicited by a ViSaGe visual stimulus generator (Cambridge Research Systems). Stimuli were presented using an M115HD projector (Dell). The following two types of visual stimuli were used to characterize responses: a full-field, spatially uniform stimulus that stepped between two radiance levels and a pseudorandom binary white-noise stimulus (Reid et al., 1997) that consisted of a 16 × 16 grid of squares that were white or black one-half of the time, as determined by an m-sequence of length 2^15^–1. The spectral radiance was maximal at 450, 515, and 630 nm; measured 170, 60, and 129 μW/sr/m^2^/nm, respectively, during full-field light onset; and was <0.5 μW/sr/m^2^ between 380 and 760 nm during full-field light offset (model 670 SpectraScan, Photograph Research). RGC light responses were identified as On or Off by reverse correlation analysis of the response to m-sequence stimulation (Reid et al., 1997), and as transient or sustained based on whether the response diminished or not during steps of full-field illumination (Cleland et al., 1971). In experiments that tested whether spikes could be activated electrically after blocking synaptically mediated light responses, the superfusate was switched from Ames medium to Ca^2+^- and Mg^2+^-free Ames medium (A1372-25A, United States Biologicals) that was supplemented with 0.1 mm Ca^2+^ and 3.4 mm Mg^2+^ (or, in a few experiments, 0.5 mm Ca^2+^ and 3.0 mm Mg^2+^).

Offline Sorter (Plexon; RRID:SCR_000012) was used to sort the MEA-recorded waveforms based on their first three principal components. The waveforms were evaluated without *post hoc* filtering. Clusters were identified by using the expectation-maximization cluster algorithm in Offline Sorter and then manually edited for clustering errors. None of the spikes attributed to single spiking units in this study occurred simultaneously with other spikes (i.e., none showed amplitude multiplication attributable to coincident firing by more than one unit) or during a time period assumed to be the absolute refractory period (1 ms).

### *In vivo* optic chiasm, optic nerve, and optic tract recording

Optic chiasm and optic nerve spikes were recorded *in vivo* by protocols described previously (Freeman et al., 2008) and modified as noted here. The head of each anesthetized animal was supported in a stereotaxic apparatus, and a craniotomy was performed at bregma. A tungsten-in-glass microelectrode was inserted in a metal guide needle, which doubled as the reference electrode, and the recording and reference electrode leads were connected to a high-input impedance amplifier (Xcell-3x4, FHC). For optic chiasm recordings, the microelectrode was blindly lowered into the brain at or near bregma until activity was detected in response to visual stimuli (Freeman et al., 2008). For optic nerve recordings, brain tissue was removed and the microelectrode was lowered under visual guidance into the optic nerve near the chiasm (Partida et al., 2018). Signals were amplified differentially (10,000×), bandpass filtered (second-order Butterworth filter, 10 Hz to 1 kHz), and digitally sampled at 100 kHz. (High-pass filtering at 10 Hz did not alter spike duration and was used to reduce slow drift in our recording baselines.) Light responses of individual RGCs having well isolated spikes were classified as On, Off, sustained, and transient using a battery of tests, as described previously (Freeman et al., 2008; Heine and Passaglia, 2011). Data were then collected for constant full-field illumination (30 cd/m^2^) or, if maintained discharge was low, for alternating step increases to 60 cd/m^2^ and decreases to 0 cd/m^2^ with a repeat period of 1 s. Stimuli were displayed on a video monitor measuring 30.2 × 40.4 cm (Trinitron GDM-C520, Sony), running at 100 Hz, and viewed at a distance of 16.5 cm from the cornea. The spectral radiance was maximal at ∼455, 520, and 630 nm, and measured at 767, 955, and 1177 μW/sr/m^2^/nm, respectively, at 60 cd/m^2^ (model STS-VIS, Ocean Optics).

The optic tract spikes included here were recorded *in vivo* by protocols described in detail previously (Sincich et al., 2007). The spikes were recorded extracellularly by use of Epoxylite-coated tungsten microelectrodes (5–8 MΩ; Frederick Haer). The potentials were analog filtered between 0.3 and 3 kHz, differentially amplified 1000× (model 1800, A-M Systems), and acquired digitally at 25 kHz (Power1401, Cambridge Electronic Design). The shapes of individual optic tract spikes were similar to those in previous studies (McIlwain, 1964). Moreover, previous studies have shown that spike durations as brief as those we show for optic tract are not significantly distorted by the high-pass filtering used here (Vigneswaran et al., 2011).

### Somatic and axonal spike durations

We examine here the duration of extracellularly recorded spikes. To characterize the spike duration of single cells and to compare these with spikes of other cells, spike properties were typically calculated from mean waveforms. In the absence of a unique definition, and because the numbers of spikes we were able to record varied from cell to cell, we calculated spike duration in several ways.

**Figure 1.**
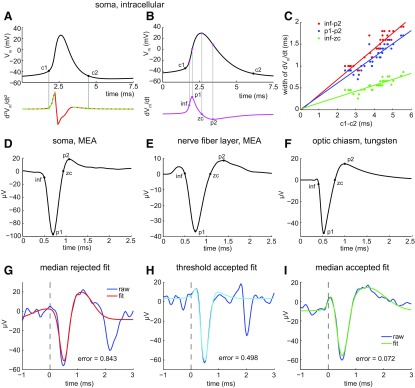
Duration, width, and shape of RGC spikes. ***A***, ***B***, Mean of cardinal spikes elicited by injection of pseudorandomly fluctuating current and recorded with whole-cell patch-clamp electrodes. ***A***, Top and bottom traces show *V*_m_ and second-order time derivative of *V*_m_ (d^2^*V*_m_/dt^2^), respectively, of an RGC soma versus time. Duration measured from c1 (base of depolarizing phase) to c2 (end of repolarizing phase). Red line plots d^2^*V*_m_/dt^2^. Green dashed lines before and after spike peak show Gaussian fits used to calculate c1 and c2, respectively, and are plotted over the red line. ***B***, Top and bottom traces show *V*_m_ and d*V*_m_/dt, respectively, versus time. Different RGC soma than in ***A***. Points labeled inf, p1, zc, and p2 mark moments when spike rises, depolarizes most quickly, reaches the peak of depolarization, and hyperpolarizes most quickly, respectively. Each time point calculated as explained in Materials and Methods. ***C***, Spike widths, and parts thereof, calculated from inf–p2 (red dots), p1–p2 (blue dots), and inf–zc (green dots), and plotted versus spike duration (c1–c2). Each dot is the mean of the widths of all spikes recorded from a single soma. Lines are linear regressions. The *y*-intercepts range between −20 and 20 μs. ***D***, ***E***, Spikes recorded on MEA and identified as somatic by biphasic waveform (***D***) and as axonal by triphasic waveform (***E***). ***F***, Spike recorded by tungsten-in-glass microelectrode from optic chiasm. Each trace (***D–F***) is the mean of all spikes recorded from each unit. ***G–I***, Best fittings of sum-of-three-Gaussians function to spike waveforms. Examples of fittings that are rejected (***G***, red) versus accepted (***H***, light blue; ***I***, green) as fits to unaveraged, MEA-recorded axonal C spikes (dark blue). The error of fit in each panel was calculated as described in Materials and Methods, and accepted if it was <0.500. Fits to the second outward peak of some spikes were interrupted by follower spikes (e.g., ***H***).

For illustrative purposes, we first measured the duration of intracellularly recorded somatic spikes as the time elapsing between two “corner” points (Fig. 1*A*, c1, c2). To locate these points, we calculated the second-order time derivative (d^2^*V*_m_/dt^2^) of the mean of the intracellular spike trace (to track how rapidly the membrane voltage changed) and then set criterion levels along the resulting waveform by comparison with studies of voltage-gated Ca^2+^ current flow during action potentials (Sabatini and Regehr, 1999). Because of noise in the d^2^*V*_m_/dt^2^, the rising phase (baseline to first peak) and the falling phase (second peak to baseline) were each separately fit with a Gaussian function centered on the peak. Only the left half or the right half of the Gaussian, respectively, was used in this fit. c1 was defined as the first point of the rising phase (left half) Gaussian fit to exceed 5% of the height of the peak of the fit. c2 was defined as the last point of the falling phase (right half) Gaussian fit to exceed 50% of the peak of the fit (Fig. 1*A*). Similar values were obtained for individual cells by fitting the rising and falling phases of the average of all spikes recorded and by averaging the durations based on fits to the rising and falling phases of each spike recorded.

Because the c1–c2 time is not available from extracellular recordings, and because the extracellular voltage is approximately the first-order time derivative (dV/dt) of the intracellular voltage, we calculated spike widths using four other time points in the d*V*_m_/dt of the mean intracellular spike waveforms. In temporal order, we refer to these points as the inflection (inf), peak 1 (p1), zero crossing (zc), and peak 2 (p2). The timing of p1 is defined as the maximum of the d*V*_m_/dt waveform. The timing of inf is defined as the maximum of the second derivative of the d*V*_m_/dt waveform in the 1 ms preceding p1. The timing of zc is an interpolated point defined as the first zero crossing following p1 (as d*V*_m_/dt returned from p1 and crossed the baseline to begin the downward deflection). The timing of p2 is defined as the minimum point of the d*V*_m_/dt waveform within the 2 ms following p1. Figure 1*B* shows these points calculated from the d*V*_m_/dt of a mean intracellular waveform (and then projected onto that mean waveform). Plots of the times that elapse between these points (Fig. 1*C*) show that the rising phase of each spike (inf–zc), the width between the moments at which the rates of depolarization and repolarization are maximum (p1–p2), and the longest width we extracted from the d*V*_m_/dt traces (inf–p2) all increase with the duration defined as c1–c2. The *r*^2^ of these lines is 0.913 for inf–p2, 0.903 for p1–p2, and 0.840 for inf–zc. This suggests that the time courses of the extracellular waveform measured by the time points defined here are linearly related to the time course of the intracellularly recorded spikes.

Extracellularly recorded spike durations were calculated from a mean waveform obtained by averaging all of the spikes collected from a given cell under a condition of interest and then finding time points comparable to those used to measure intracellularly recorded spike widths (inf, p1, zc, and p2; Fig. 1*D–F*). Inf was the time of the minimum of the second derivative of the spike waveform in the 1 ms preceding p1; p1 and p2 were the minimum and maximum voltages reached during the spike waveform (i.e., the voltages at the peak of the inward and outward deflections); and zc was the first zero crossing following p1. When constructing histograms of spike widths, if the spike numbers and frequencies were not sufficient to create a reliable mean waveform for a given time bin, we calculated the spike duration from a fitted version of each individual spike. In these cases, each spike was modeled by a sum-of-three-Gaussians function, with each Gaussian corresponding to a different region of the spike. These Gaussians were of the following form:

$$y=a\;+\;b\;*\;e^{-\left(\frac{{\left(x-c\right)}^{2}}{2*d^{2}}\right)}$$

where *a* is the *y*-offset, *b* is the height, *c* is the *x*-location of the peak, and *d* is a sharpness factor. For extracellularly recorded axonal spikes, the first Gaussian was a positive-going Gaussian fitting the peak at ∼0.4 ms, the second Gaussian was a negative-going Gaussian fitting the peak at ∼0.75 ms, and the third Gaussian was a positive-going Gaussian fitting the peak at ∼1.4 ms. The fit was accepted if there was a zero crossing in the fit between the second and third Gaussians, and the value (mean squared error of the fit)/(absolute value of the height of the second peak raised to the 1.25 power) was <0.5 (Fig. 1*G–I*). The exponent (1.25) in the denominator was hand chosen to make the fit criterion approximately equal by eye across the range of observed spike amplitudes. Without this exponent, attempts at choosing a criterion were either too permissive for low-amplitude spikes or too restrictive for high-amplitude spikes. By these criteria, ∼70% of the fits were accepted for duration calculations. Rejected fits (e.g., Fig. 1*G*) differed from accepted fits (Fig. 1*H*,*I*) most notably in the shape and amplitude of the initial outward and large inward peaks of the recorded waveforms. All fitting was done with the “fmincon” function in MATLAB (R2014A, MathWorks; RRID:SCR_001622), which allows constraints to be placed on individual parameters of the fit.

### Experimental design and statistical analysis

We compared the durations of spikes generated by single units and of spikes generated by different units (see below). One comparison contrasted the duration of spikes as a function of the time between each spike and the spike that preceded it (i.e., the interspike interval or “quiet period”). We will refer to the first spike after a long interspike interval as a cardinal (“C”) spike and to spikes following shorter interspike intervals as follower (“F”) spikes (Partida et al., 2018). To determine the minimum interspike interval that we used to define C spikes in this study (50 ms), we compared C spikes that were preceded by quiet periods of different lengths. For each cell, we found all C spikes that fell within bins of quiet periods ranging from 50 to 150 ms, 150 to 300 ms, 300 to 500 ms, 500 to 1000 ms, and 1000 to 2000 ms. Cells with at least five spikes in each of these five bins were included for further analysis (nerve fiber layer, *n* = 56; optic chiasm, *n* = 26). For each of these cells, a mean spike was created from the individual spikes in each bin, and the duration of each mean spike was calculated as described above. We then used interspike intervals that allowed C spike durations to recover from the effects we are reporting here. Having defined C spikes, we compared C and F spikes at interspike intervals found during light responses (Heine and Passaglia, 2011; Baden et al., 2016).

To compare the duration of C and F spikes, we plotted the duration of F spikes as a function of C spike durations. We also plotted histograms of the percentage increase in F1 spike duration (i.e., the duration of the first F spike), calculated by normalizing the Euclidean distance of the C/F spike width pairs (inf–p2) from the unity line by the mean of the C spike durations for individual somata and axons. These normalized C–F changes in width were histogrammed from −40% to 40% in bins of 1% (for optic chiasm data) or 5% (all other data).

We compared spike durations between RGCs that responded differently to illumination (see Results; Cleland and Levick, 1974; Fukuda and Stone, 1974). Because we found cell-to-cell differences in the number of spikes generated, we digitally averaged the cardinal spikes of a selected cell and calculated the duration of the averaged waveform. We then repeated this calculation for each of the other cells of the same type, yielding one duration per spiking unit. The mean of the durations of these units, and the digital average of the averaged waveforms, were taken as representative of the cell type. This enabled us to compare the spike durations of different cell types without biasing the mean waveform by large numbers of spikes from a few cells with uncharacteristically narrow or broad spikes.

Likewise, each cell generated different numbers of spikes in the dark and light, and these durations could vary from cell to cell. To test whether spike duration differed in the dark and light, we recorded spikes in response to two changes in illumination (dark–light, light–dark) and compared the duration of equal numbers of spikes in dark versus light prior to these transitions and also in dark versus light after these transitions. To do so, we (1) calculated the mean of the durations of all spikes from a given unit during a given time bin in the light (to compare spikes before the transitions) and in the dark (to compare spikes after the transitions); (2) identified the condition that produced fewer spikes (e.g., dark for an On RGC); (3) equalized the number of spikes to be compared by randomly selecting the same number of spikes under the other condition (e.g., light for that On cell); (4) normalized the duration of each of the raw spikes from step 2 and each of the selected spikes from step 3 by the mean from step 1 for the time bin under consideration; (5) repeated steps 1–4 for all units recorded from; (6) pooled the normalized durations from these units; (7) calculated the mean of these normalized durations; and (8) repeated steps 1–7 for each time bin. We then plotted the mean of the normalized durations as a function of time before and after the dark–light and light–dark transitions.

Statistical analyses were performed using MATLAB (R2014A). Unless stated otherwise, measurements are presented as the mean ± SEM and sample size. For sufficiently large sample sizes, we used two-tailed Student’s *t* tests to assess whether differences are statistically significant and report the mean, SEM, value range, sample size, and *p* value. For the data presented in scatter plots, we used Wilcoxon signed-rank tests to assess whether differences are statistically significant, plot the mean and SEM, and state the sample size and *p* values. The sample size (i.e., the number of spiking somata or axons) are stated in the text and figure legends.

## Results

This study compares the duration of spikes that are recorded from rat nerve fiber layer, optic nerve, and optic chiasm. To test whether and how these spikes differ, we record spikes (1) as they fire spontaneously, (2) during responses to electrical stimulation, and (3) before and after the onset and cessation of illumination. We then measure spike duration as the time that elapses between pairs of time points (defined in the Materials and Methods as p1, p2, inf, and zc) during each spike waveform (Fig. 1) and compare spikes that fire at different frequencies and in functionally different cell types. When describing multiple spikes, we refer to the first spike after a long interspike interval as a cardinal (C) spike and to spikes after shorter interspike intervals thereafter as follower (F) spikes (Partida et al., 2018). Additionally, to test whether spikes broaden in other species, we compare C and F spikes that were recorded in rhesus macaque optic tract (Sincich et al., 2007). The comparisons below are based on C spikes that were each preceded by a ≥50 ms spike-free period, and on the first F (F1) spike that fired within 30 ms after each C spike, unless stated otherwise.

### Activity alters intraretinal spike duration but not extraretinal spike duration

Voltage-gated Na^+^ current declines in amplitude as RGCs are depolarized at frequencies >10 Hz (Hidaka and Ishida, 1998; Kim and Rieke, 2003; Hayashida and Ishida, 2004). The types of K^+^ current that gate at the voltages traversed by RGC spikes also inactivate during sustained (Lipton and Tauck, 1987) and repeated (Geiger and Jonas, 2000; Faber and Sah, 2003) depolarizations. These activity-induced losses of current would be expected to reduce spike maximum rate-of-rise and amplitude (Fleidervish et al., 1996) and increase spike duration (Jackson et al., 1991; Giese et al., 1998). We therefore tested whether a spike preceded recently by another spike (short interspike interval) differed in shape from spikes preceded by long spike-free periods (long interspike intervals).

**Figure 2.**
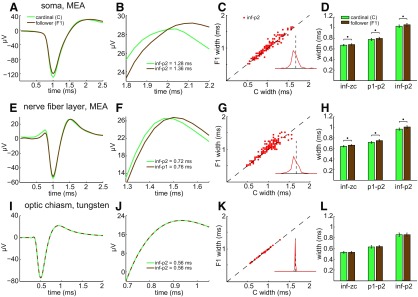
Activity-induced broadening of intraretinal, but not extraretinal, F1 spikes. ***A***, ***E***, ***I***, Averages of C spikes (green) and of F1 spikes (brown) recorded from an RGC soma (***A***), nerve fiber layer axon (***E***), and optic chiasm axon (***I***), and analyzed as in Figure 1*D–F*, respectively. ***A***, ***E***, ***I***, Mean spikes aligned horizontally to p1 peaks. ***B***, ***F***, ***J***, Expanded views of p1–p2 from ***A***, ***E***, and ***I***, respectively. Lines in ***I*** and ***J*** are dashed to show the overlap of C and F1 spikes. ***C***, ***G***, ***K***, Scatter plot of F1 spike width versus C spike width for all intraretinal somata (***C***; *n* = 166), all intraretinal axons (***G***; *n* = 201), and all optic chiasm axons (***K***; *n* = 38) recorded from. Unity line plots where ***C*** and ***F*** spike durations would match, and insets are histograms of the percentage increase in F1 spike duration. The dashed vertical line in each inset corresponds to the unity line in the scatter plot; line height corresponds to 70% of the total count of spiking units at each recording locus. ***D***, ***H***, ***L***, Bar plots (mean ± SEM) compare C (green) and F1 (brown) spike widths plotted in ***C***, ***G***, and ***K***, respectively, and were measured by the indicated time points. Asterisks are centered above the mean widths that differ by statistically significant amounts.

Because our MEA recordings captured somatic and axonal spikes, we first checked whether repeated spike firing altered somatic spikes as reported previously (O’Brien et al., 2002; Weick and Demb, 2011). Repetitive spiking broadened spikes we recorded from somata (*n* = 166) *in situ* on multielectrode arrays (Fig. 2*A–D*). Plotting F1 spike duration versus C spike duration shows that most F1 spikes were broader than the C spikes that preceded them. Replotting these data as a histogram of the activity-induced changes in F1 spike durations shows that a large majority of the F1 spike durations are longer than the C spike durations (Fig. 2*C*, inset). The differences in duration were statistically significant regardless of the time points they were measured from (Fig. 2*D*; *p* = 5.68e-4, 0.0101, and 8.34e-4 for inf–zc, p1–p2, and inf–p2, respectively). These effects were rapid in onset and reversible, in that they were seen at interspike intervals as short as 3 ms and because C spikes presented a consistent (control) shape after spike-free periods of ≥30 ms (see below).

**Figure 3.**
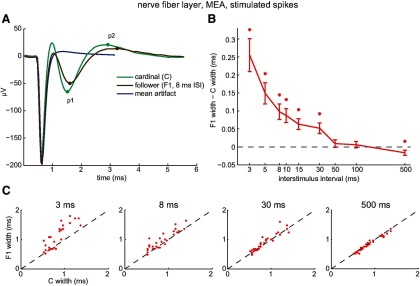
Activity-induced broadening of electrically elicited nerve fiber layer spikes. Stimulating and recording MEA electrodes separated by ≥400 μm. ***A***, C (green) and F1 (brown) spikes elicited by current pulses separated by 8 ms. Stimulus artifact (blue) after suppressing spikes by repeated stimulation. ***B***, Mean ± SEM of differences between C and F1 spike widths as a function of interstimulus interval. Spike widths were calculated as the time between p1 and p2 (e.g., dots on spike waveforms in ***A*** after the stimulus artifact has faded). Asterisks positioned above statistically significant differences. ***C***, F1 spike widths as a function of C spike widths at interstimulus intervals ranging from 3 to 500 ms. Each data point plots the mean C and F1 spike widths of an individual axon.

Do short interspike intervals also broaden spikes in axons? We examined this first in the nerve fiber layer, and thereafter in the optic nerve, optic chiasm, and optic tract. Averaged across all of the intraretinal axons we recorded from (*n* = 201), short interspike intervals increased the width of spontaneously firing F spikes (Fig. 2*E–H*). The increase was statistically significant (Fig. 2*H*) regardless of whether spike width was measured as inf–zc (*p* = 4.14e-7), p1–p2 (*p* = 4.61e-5), or inf–p2 (*p* = 4.72e-6). Aligning the mean waveforms of C and F1 spikes at their p1 peaks showed that activity increased the time elapsing between p1 and p2 (Fig. 2*F*). The F1 spike width was broader than the C spike width in most of the nerve fiber layer axons we recorded from (Fig. 2*G*, inset therein). Similar effects were produced by electrically activating C and F1 spikes with an MEA electrode and varying the interval between the pulses that elicited these spikes (Fig. 3). The p1–p2 width increased (Fig. 3*A*) for nearly all of the nerve fiber layer axons we recorded from (e.g., Fig. 3*C*, 3 ms panel) and the increases in spike width were statistically significant at time intervals between 3 and 30 ms [Fig. 3*B*; intervals for *p* values are in parentheses: 7.24e-5 (3 ms), 2.28e-5 (5 ms), 8.61e-5 (8 ms), 8.40e-5 (10 ms), 2.17e-4 (15 ms), 0.0015 (30 ms), 0.3989 (50 ms), 0.5017 (100 ms), 0.0268 (500 ms)]. Figures 2 and 3 thus show that short interspike intervals broaden the spikes of RGC somata and intraretinal axons.

Do short interspike intervals also broaden spikes in extraretinal axons? We examined this by recording from optic chiasm (and, to the extent we found possible, optic nerve) and by analyzing spikes recorded from optic tract. We detected no effect of activity on optic chiasm F spike widths (Fig. 2*I–L*; *p* = 0.8851, 0.4463, and 0.4405 for inf–zc, p1–p2, and inf–p2, respectively). Scatter plots showed no cases where F1 and corresponding C spike widths differed (Fig. 2*K*; *n* = 38). Although we were unable to record for periods long enough to characterize optic nerve spikes in detail, we obtained comparable results from a limited number of optic nerve axons (*n* = 3; not illustrated). C and F1 axonal spikes from two different optic nerves showed no difference in duration at interspike intervals <50 ms. C and F1 spikes from one other optic nerve increased in duration at interspike intervals <30 ms. These results show that repetitive spike firing broadens spikes in intraretinal axons but not in extraretinal fibers (with one exception in our datasets).

**Figure 4.**
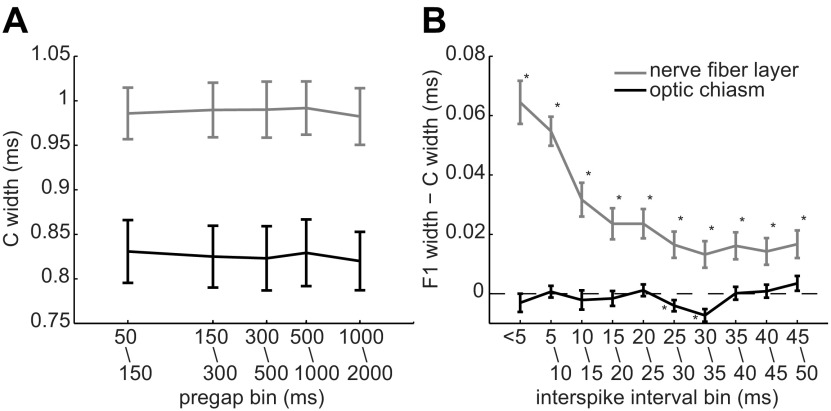
Spike widths (inf–p2) versus the timing of preceding and following spikes. Points and error bars plot the mean ± SEM of ordinate values measured in nerve fiber layer (gray; *n* = 56) and optic chiasm (black; *n* = 26) axons and were pooled over the indicated time bins. ***A***, C spike widths as a function of the spike-free period preceding each C spike. ***B***, Difference between C and F1 spike widths as a function of the interspike interval. Asterisks are adjacent to statistically significant differences.

Given that C spikes broadened intraretinal F1 spikes, we tested two potential reasons why C spikes might not have broadened extraretinal F1 spikes. One possibility is that the spike-free times used to define C spikes were long enough for extraretinal C spikes to return to control values, but were too short for intraretinal C spikes to return to control values. Our data indicate otherwise, as we found no difference between C spike inf–p2 widths measured after quiet periods between 50 and 2000 ms, for axonal spikes recorded either intraretinally or extraretinally (Fig. 4*A*). As determined by one-way ANOVA, there were no significant differences between these C spike widths in the nerve fiber layer (*F*_(4,275)_ = 0.38, *p* = 0.8195) or in the optic chiasm (*F*_(4,125)_ = 0.02, *p* = 0.9995). A second possibility is that the extraretinal C and F1 spikes in our recordings were separated by longer times than the intraretinal spikes. Again, our data indicate otherwise, as plots of inf–p2 showed that the broadening of nerve fiber layer F spikes increased as the interspike interval decreased from 35 to ≤5 ms and that optic chiasm F spikes did not broaden over the same range of interspike intervals (Fig. 4*B*). These results thus show that extraretinal spike widths are not altered by interspike intervals that broaden spikes in intraretinal axons.

**Figure 5.**
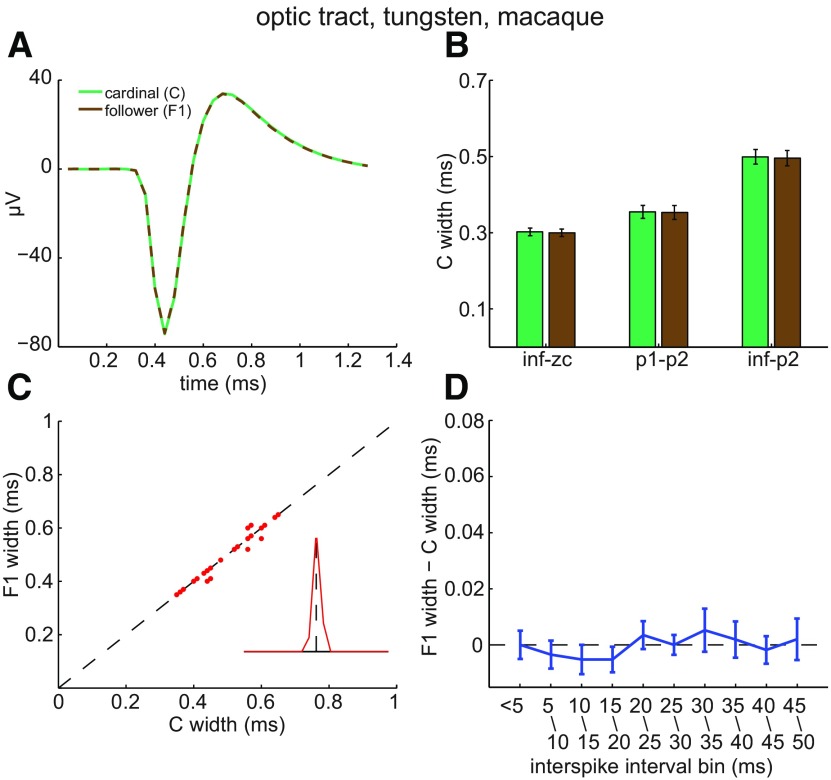
Lack of activity-induced broadening of optic tract spikes. ***A***, Averages of C spikes (green) and of F1 spikes (brown) recorded from an optic tract axon, plotted by dashed lines and aligned horizontally to their peaks. The traces are indistinguishable in amplitude and time course. ***B***, C (green) and F1 (brown) spike widths (mean ± SEM) measured by indicated time points for each optic tract axon and then averaged across all axons recorded from (*n* = 23). ***C***, C and F1 spike widths for each optic tract axon recorded from. In several instances, dots plotting C and F1 values from multiple fibers overlapped. If only two data points overlapped, one was displaced by ±0.01 ms vertically and laterally. In the few cases where three data points overlapped, points were plotted at (*x*, *y*), (*x* + 0.01, *y* + 0.01), and (*x* − 0.01, *y* − 0.01). These displacements prevented the data from being moved relative to unity line and allowed the figure to show data from all fibers. ***D***, The difference (mean ± SEM) between axonal C and F1 spike widths as a function of interspike interval. None of the mean differences were statistically significant.

The results in Figures 2–4 raise two questions. First, does spike broadening occur distal to the optic chiasm? Second, do short interspike intervals broaden spikes in RGC axons of other species? We addressed both questions by analyzing spikes recorded from rhesus macaque optic tract fibers (*n* = 23; Sincich et al., 2007). Figure 5 plots averages of the C spike waveforms and of the F1 spike waveforms recorded from an individual fiber (Fig. 5*A*), C and F1 spike widths averaged across all of the fibers recorded from (Fig. 5*B*), F1 spike width as a function of C spike width for each of the fibers recorded from (Fig. 5*C*), the Euclidean distance of the paired C/F values from the unity line (Fig. 5*C*, inset), and the difference between C and F1 spike width as a function of interspike interval (Fig. 5*D*). These panels in Figure 5 show that C spikes did not induce F spike broadening in optic tract (*p* = 0.1177, 0.6647, and 0.4264 for inf–zc, p1–p2, and inf–p2, respectively) and that, in this respect, the results we obtained from macaque optic tract closely resembled those we obtained in rat optic chiasm.

### Light alters intraretinal spike duration but not extraretinal spike duration

**Figure 6.**
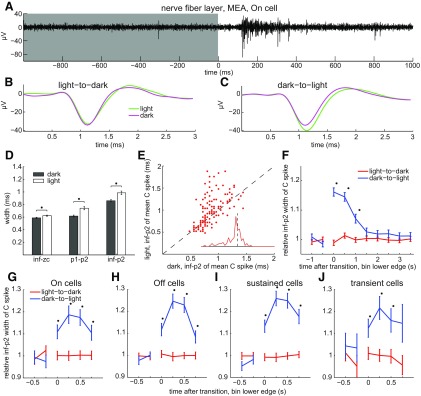
Light-induced broadening of intraretinal axonal C spikes. ***A***, Spikes of transient On cell, identified by low spike frequency in darkness (e.g., single spike at *t* ∼ −300 ms in ***A***) and brief increases in spiking following light onset (top trace, single unaveraged sweep). Gray and white background behind spikes show timing of dark and light, respectively. ***B***, ***C***, Average of spikes recorded before and after light offset (***B***) and before and after light onset (***C***), per color code. ***D***, Spike widths calculated from indicated time points and averaged across all axons recorded from in dark versus light (mean ± SEM; *n* = 435 axons for inf–zc; *n* = 212 axons for p1–p2 and inf–p2). ***E***, Mean spike inf–p2 width in light versus dark, where each dot plots values of a different axon. Unity line plots where C spike durations in light would match those in dark. Inset, Euclidean distances from unity line of points in ***E***. ***F***, Mean ± SEM of normalized spike widths before and after onset (blue) and offset (red) of light (*n* = 136 axons). Spike durations pooled over 500 ms time bins. ***G–J***, Normalized spike width as function of time for On (***G***), Off (***H***), sustained (***I***), and transient (***J***) cells. Spike widths pooled over 250 ms time bins. ***F–J***, Spike width of each axon during each time bin normalized to the mean of all C spike widths of that axon in the dark. Lines connect values in dark or in light, but not at transition between dark and light. Asterisks are next to statistically significant differences between dark and light values.

The results in Figure 4*A* indicate that, given sufficiently long quiet periods, axonal C spike widths are constant. However, these spikes were recorded under constant illumination, and RGCs normally respond to changes in illumination. Because alternating steps of light and dark modulate spike firing frequency in all functional types of RGCs identified to date (Baden et al., 2016), we next tested whether the onset of light and/or dark alter axonal C spike shape. To assess this intraretinally, spikes were recorded on MEAs. Light onset increased spike firing frequency (i.e., elicited On responses; Hartline, 1938) at some electrodes (Fig. 6*A*). None of these light responses were sluggish in onset and offset as in intrinsically photosensitive RGCs (Berson et al., 2002). When we sorted and averaged the spike waveforms recorded during these responses (i.e., during 1.0 s before and after light offset, and during 1.0 s before and after light onset), the resulting waveforms showed that (1) after light onset, spike width was greater during illumination than during each preceding dark period (Fig. 6*C*); and (2) spike width did not noticeably change after light offset (i.e., it did not differ between light periods and subsequent dark periods; Fig. 6*B*).

**Figure 7.**
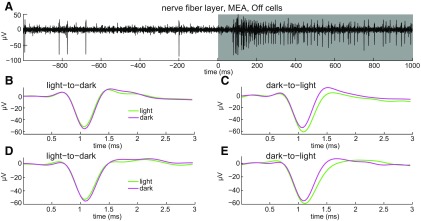
Light-induced broadening of intraretinal axonal C spikes. ***A***, Spikes of Off axons identified by the increase in spiking following light offset (top trace, continuous recording, single unaveraged sweep). ***B–E***, Spikes recorded on MEA, identified as axonal by triphasic waveform. Format is as shown in Figure 6. ***B***, ***C***, Plots of mean of spike waveforms extracted by PCA from one axon before and after light offset (***B***) and before and after light onset (***C***), per color code. ***D***, ***E***, Plot mean of spike waveforms extracted by PCA from another axon for same transitions between dark and light, and formatted as in ***B*** and ***C***.

Because light increased both spike frequency and spike width in Figure 6*A*,*C*, and *D*, we next asked whether illumination broadens spikes simply by increasing spike frequency. We found that the effects of light and short interspike intervals on spike width differ in multiple ways. One is that while short spike intervals increase F spike width (Figs. 2–4), light onset increases the width of all spikes, including C spikes (Fig. 6*C*,*D*). A second difference is that light can increase spike width even if it decreases spike frequency. For example, although spike frequency is less during light than during dark during the Off light response in Figure 7*A*, the spike width is greater during light (Fig. 7*C*). Notably, spikes from three axons were extracted by principal component analysis (PCA) in this recording and light onset increased the mean spike width in all three (e.g., Fig. 7*C*,*E*). Moreover, in RGCs that generated either On or Off light responses, short interspike intervals increased F spike width both in the light and the dark. These results (Figs. 6, 7) show that light and short interspike intervals can modulate intraretinal RGC spike duration independently and suggest, in turn, differences in underlying mechanisms.

To compare the spike widths of different axons regardless of differences in the numbers of spikes they generated, we averaged all of the spikes recorded from each axon in the light and in the dark. Across all of the axons we recorded from, the mean of these averages during the first 1.0 s after light onset was ∼15% greater than during the first 1.0 s after light offset, and the differences of these means were statistically significant (Fig. 6*D*, asterisks; *p* = 0.0025, 2.8e-08, and 2.1e-07 for inf–zc, p1–p2, and inf–p2, respectively). Viewed axon by axon, the C spike widths in the light were greater than those in the dark for most of the axons we recorded from and the largest changes in C spike width we found during illumination were increases (Fig. 6*E*, and inset). Plotting the averages of the C spike widths as a function of time before and after the change in illumination shows that C spike width increased markedly at light onset, remained high during the first second thereafter, and then declined during the subsequent 1–1.5 s to values that did not differ by statistically significant amounts from spike durations in the dark (Fig. 6*F*, blue lines). This plot also shows that C spike width did not noticeably change after light offset (i.e., it did not differ between the light periods and subsequent dark periods; Fig. 6*F*, red lines).

Does illumination broaden spikes in all axons? Although light significantly increased the mean spike width, some axons we recorded from showed either no change in width or a decrease in width (Fig. 6*E*). This might reflect systematic differences between certain subtypes of RGC or variability among axons regardless of type (e.g., in the rate of return to control values). Figure 6*G–J* summarizes the spike widths we measured in axons classified into four types (On, Off, sustained, and transient) for comparison with previous studies (Fukuda and Stone, 1974; Wong et al., 2012). These plots show that light onset increased spike width by statistically significant amounts in each cell class and also illustrates differences with the effects of short interspike intervals in addition to those noted above. First, the increases during illumination were a few fold larger than the effect of C spikes on F1 spikes (compare Figs. 4*B*, 6*F–J*). Second, spike width increased gradually within the first 500 ms after light onset (Fig. 6*G*–*J*) but increased maximally within 2–5 ms after C spikes (Fig. 4*B*). This became apparent when we pooled the duration data in 250 ms bins (Fig. 6*G–J*) but not in 500 ms bins (Fig. 6*F*). Altogether, the results in Figures 6 and 7 show multiple differences between the effects of light and interspike interval on RGC spike duration.

**Figure 8.**
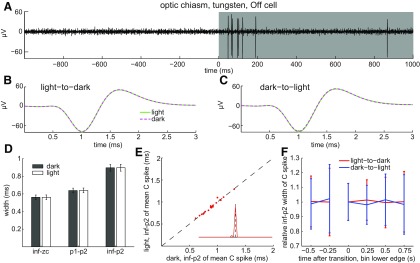
Absence of light-induced broadening in optic chiasm C spikes. ***A***, Spikes of transient Off cell identified by spike burst after light offset (top trace, single unaveraged sweep, formatted as in Fig. 6). ***B***, ***C***, Averages of spike waveforms recorded before and after light offset (***B***) and before and after light onset (***C***). ***D***, Spike widths averaged across all fibers recorded from. ***E***, Single-fiber C spike widths in light versus dark. Scatter plot and inset constructed as in Figure 6. ***F***, Change in mean C spike width as a function of time before and after light onset (blue) and offset (red). Values pooled across all optic chiasm axons recorded from (*n* = 19 for inf–zc, p1–p2, and inf–p2) and formatted as in Figure 6. Spike widths pooled over 250 ms time bins.

Having found that light broadens spikes in intraretinal axons, we next asked whether light broadens spikes in extraretinal axons. Averaged across all of the optic chiasm axons we recorded from (*n* = 19), none of the mean C spike widths measured in darkness differed by significant amounts from those measured in light (Fig. 8*D*; *p* = 0.9536, 0.8184, and 0.9369 for inf–zc, p1–p2, and inf–p2, respectively). None of the mean C spike widths differed between dark and light by >100 μs (Fig. 8*E*), and the inf–p2 widths in the dark and light showed no significant differences as a function of time before and after the changes in illumination (Fig. 8*F*). Thus, in marked contrast to the effects we found in nerve fiber layer axons (Figs. 6, 7), we found no differences between C spike durations in the dark and light in the optic chiasm (Fig. 8). Together, Figures 4-8 show that light and short interspike intervals broaden the spikes of intraretinal RGC axons but that neither light nor short interspike intervals broaden spikes of extraretinal RGC axons.

### Cell subtype-specific spike duration differences are preserved in intraretinal and extraretinal axons

Spikes of different duration have been recorded intraretinally from some subtypes of RGC. For example, RGCs termed “transient” and “sustained” generate brief and longer bursts of spikes following the onset of illumination (Cleland et al., 1971), and electrical stimulation elicits somatic spikes of shorter duration in transient cells than in sustained cells (Fukuda and Stone, 1974). Likewise, light onset and offset elicit increases in spiking in RGCs termed “On” and “Off,” respectively, and morphologic counterparts of these cells have been found to generate individual somatic spikes of shorter duration in Off cells than in On cells (Wong et al., 2012). We therefore compared axonal C spikes of these types of RGCs, and did so in intraretinal and extraretinal axons.

**Figure 9.**
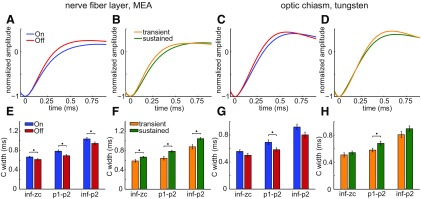
C spike widths of RGC axons distinguished by light response. Mean spike waveform of each cell type calculated by aligning all C spikes of a given axon to their p1 time point and averaging them; repeating this for the C spikes of all axons of a given cell type; and then calculating the mean of all the averaged waveforms. ***A–D***, Each panel superimposes the portion of the mean spike waveforms compared, from just before p1 until just after p2. These are color coded as indicated and aligned to their p1 time points (as in Fig. 2). ***E–H***, Widths of all C spikes recorded from each axon calculated and averaged from time points listed below each pair of bars. Bars (color coded as indicated) plot the mean ± SEM of averages from all axons of On and Off units in nerve fiber layer (***E***) and optic chiasm (***G***), and of transient and sustained units in nerve fiber layer (***F***), and optic chiasm (***H***). Sample sizes are 85 On, 80 Off, 44 transient, and 110 sustained axons in nerve fiber layer, and 19 On, 19 Off, 15 transient, and 22 sustained axons in optic chiasm. Asterisks are above statistically significant differences.

The C spikes were broader in On nerve fiber layer axons than in Off axons (Fig. 9*A*,*E*). The difference between the mean widths of these On and Off units was ∼10% and was statistically significant (*p* = 0.0359, 0.0124, and 0.0328 for inf–zc, p1–p2, and inf–p2, respectively; *n* = 85 On units and *n* = 80 Off units). Likewise, the C spikes of On optic chiasm axons (*n* = 19) were ∼15% broader than those of Off optic chiasm axons (*n* = 19; Fig. 9*C*,*G*). This difference was statistically significant when calculated from p1–p2 (*p* = 0.0174). The same trend was seen when the spike widths were calculated as inf–zc or inf–p2. However, the differences did not reach statistical significance (*p* = 0.1375 and 0.0538, respectively).

The intraretinal axonal C spikes of sustained cells (*n* = 110) were ∼19% broader than those of transient cells (*n* = 44; Fig. 9*B*,*F*). These differences were statistically significant (*p* = 0.0046, 7.77e-4, and 5.70e-4 for inf–zc, p1–p2, and inf–p2, respectively). Likewise, the C spikes of sustained optic chiasm axons were broader than those of transient units (Fig. 9*D*,*H*). Calculated as p1–p2, the spikes of the sustained units (*n* = 22) were ∼11% broader than those of the transient units (*n* = 15), and this difference was statistically significant (*p* = 0.0412). The spike widths calculated from other time points (inf–zc, inf–p2) were also greater in sustained units than in the transient units, although these differences did not reach statistical significance (*p* = 0.3391 and 0.1655, respectively).

These results (Fig. 9) show that, although extraretinal axons normalize spike duration within cell types (i.e., as spikes propagate from intraretinal to extraretinal segments), they preserve spike duration differences between cell types (i.e., between sustained and transient cells, and between On and Off cells).

## Discussion

Our results identify three properties of spikes in RGC axons. First, short interspike intervals broaden spikes in intraretinal axons but not in extraretinal axons. Second, light onset broadens spikes in intraretinal axons but not in extraretinal axons. Third, axonal spike duration differs between functionally different RGCs both intraretinally and extraretinally. Below, we compare these findings to spikes in other preparations and relate spike durations to signal transfer by RGCs.

### Spike broadening and normalization

Spike broadening in the nerve fiber layer and not in the optic chiasm distinguish axons of RGCs and other neurons in two respects. First, unlike axons that can conduct and transmit information with spikes of different shapes (Juusola et al., 2007), individual extraretinal axons eliminate analog differences in spikes and thus reduce the number of spike parameters that visual stimuli can modulate. Second, extraretinal axon segments are as far as several millimeters from the AISs that initiate RGC spikes intraretinally (Wollner and Catterall, 1986; Sekirnjak et al., 2008), and thus are much further from somata than the axonal regions that normalize spike duration in other systems (Williams and Stuart, 1999; Meeks et al., 2005; Khaliq and Raman, 2006; Kole et al., 2007; Foust et al., 2010; Popovic et al., 2011). This separation might be a “best possible” design, in which retinae use unmyelinated RGC axons intraretinally to avoid the opacity of myelin (Straatsma et al., 1979) and then compensate for the slower spike propagation in these fibers by increasing conduction velocity in the optic nerve (Stone and Freeman, 1971) and between the optic nerve and optic tract (Baker and Stryker, 1990).

We did not attempt to identify how short interspike intervals broaden spikes intraretinally, in part because the same intervals do not broaden spikes in optic chiasm or optic tract. We also do not know of any ion channel identifications or current measurements in RGCs that would explain or predict extraretinal spike normalization. However, studies of other neurons suggest possibilities, having found that the kinetics and high densities of Kv1, Kv3, and KCa1.1 channels facilitate Na^+^ current recovery from inactivation (Kole et al., 2007; Li et al., 2011; Ritzau-Jost et al., 2014) and guard against spike broadening. Increases in Na^+^ current density, and lowered membrane capacitance in myelinated axons, should also help to curb spike broadening extraretinally.

### Generality

The effects we report here of short interspike intervals and light onset on spike width are consistent with the possibility that unmyelinated and myelinated axon segments differ in ways besides conduction velocity. Two questions emerge from these results. First, do spikes broaden in unmyelinated segments of all axons at the interspike intervals examined here (Figs. 3, 4)? This would be of interest to test in several preparations, including axons that are unmyelinated during development and myelinated in adulthood (Foster et al., 1982), adult optic nerves that contain several (and, in a few cases, exclusively) unmyelinated axons (Gaze and Peters, 1961; Holmberg, 1972; Hokoç and Oswaldo-Cruz, 1978), myelinated axons with unmyelinated segments interspersed between their proximal and distal ends (Salami et al., 2003; Tomassy et al., 2014), and axons before and after demyelinating diseases (Ulrich and Groebke-Lorenz, 1983; Utzschneider et al., 1993). Second, do spikes broaden in RGC axon terminals and, if so, does this alter signal transfer? Answering these questions will require methods for testing whether spike duration and amplitude both change (Sabatini and Regehr, 1999) and for assessing the variability of Ca^2+^ signaling and spike duration at different terminal boutons even in single axon branches (Brenowitz and Regehr, 2007; Rowan et al., 2016), especially given the morphology of RGC axon terminals (Bowling and Michael, 1980; Hammer et al., 2015; Morgan et al., 2016).

A recent study found that the amplitudes of extracellularly recorded spikes are “regularized” as they propagate toward the optic disk in M1-type intrinsically photosensitive RGC axons (Milner and Do, 2017). These results differ from ours in that activity-induced increases in spike width were not noted. Whether spike broadening occurs in M1 RGCs at the high frequencies we examined here remains to be tested.

### Spike broadening at light onset

Although light significantly increased the mean spike width in our intraretinal recordings (Fig. 6*F*), this effect was transient, and some axons showed either no change in width or a decrease in width (Fig. 6*E*). These properties imply that the light-triggered increases in spike duration were not a nonspecific (e.g., photoelectric) artifact (Moody et al., 1969). Nevertheless, because short interspike intervals increase intraretinal spike duration, light onset increases spike frequency in On cells, and light offset increases spike frequency in Off cells, we were surprised to find that light onset increased spike duration in both On and Off cells (Fig. 6*G*,*H*). Because light also broadened spikes regardless of whether the spikes were C or F spikes, and the time course of light-induced broadening was much slower than that of activity-dependent broadening (Figs. 4*B*, 6*F–J*), a light-modulated synaptic input to On and Off RGCs seems more likely than short interspike intervals to have caused this broadening. How these inputs operate remains to be identified. However, previous studies have found tyrosine hydroxylase-immunopositive (Kolb et al., 1990) and GABA-immunopositive (Mosinger et al., 1986) neurites in the nerve fiber layer and GABA receptors on RGC axons (Hughes et al., 1989).

### Cell-specific spike duration differences between axons

Our results show that axonal spikes of functionally different RGCs differ in duration, and that the cell type-specific differences between nerve fiber layer axons are also found in the optic chiasm (Fig. 9). Our finding that axonal spike widths of sustained RGCs are longer than those of transient RGCs, and that axonal spike widths of On RGCs are longer than those of Off RGCs, agree with differences reported for the somata of these cell types in rats (Wong et al., 2012) and cats (Fukuda and Stone, 1974; O’Brien et al., 2002).

The spike widths of these On and Off cells, and those of the transient and sustained cells, differed by ∼15% (Fig. 9). Spike duration differences of similar magnitude have been found in other neurons, before and after activity-induced broadening, to significantly increase the amplitude of responses to neurotransmitter released by these spikes (Sabatini and Regehr, 1997; Geiger and Jonas, 2000). Thus, broader RGC spikes may transmit signals at higher efficacy than narrower spikes at synapses where single spikes elicit postsynaptic responses (Burke and Sefton, 1966; Cleland and Lee, 1985; Blitz and Regehr, 2003; Gollisch and Meister, 2008).

Do narrower spikes drive other types of synapses better than broader spikes? This would be expected to occur at retinogeniculate synapses that transmit at high efficacy if two or more spikes reach synaptic terminals within short time intervals because RGCs that generate the narrowest spikes can fire repeatedly at the highest rates (O’Brien et al., 2002; Wong et al., 2012; for review, see Bean, 2007) and because synaptic efficacy increases as instantaneous spike frequency increases to >20 Hz (Singer et al., 1972; Mastronarde, 1987; Usrey et al., 1998; Sincich et al., 2007; Weyand, 2007). For example, our finding that Off RGC axons generate narrower spikes than On RGC axons raises the possibility that Off RGCs drive thalamic neurons better than On RGCs. Similarly, our finding that transient RGC axons generate narrower spikes than sustained RGC axons raises the possibility that transient RGCs drive thalamic neurons better than sustained RGCs. The magnitudes of these differences are difficult to predict because Off RGCs have been found to conduct spikes more rapidly than On RGCs (Fukada, 1971; Vaney et al., 1981), transient RGCs have been found to conduct spikes more rapidly than sustained RGCs (Fukada, 1971; Cleland et al., 1971), and spike-induced spike slowing reduces the maximum spike frequency generated by slowly conducting RGC axons more than that of rapidly conducting RGC axons (Partida et al., 2018). However, these trends would increase the difference in signal transfer by Off versus On RGC axons, and by transient versus sustained RGC axons, and in turn contribute to the spread of maximum synaptic efficacies observed to date (Usrey et al., 1998). These differences may be even more subtype specific in the cat, in that Off brisk-transient RGC axons conduct spikes faster than On brisk-transient axons, and Off and On cells of other subtypes (brisk sustained, sluggish) do not differ in conduction velocity (Kirk et al., 1975). Similarly, macaque Off midget RGCs generate narrower spikes than On midget RGCs, and the spike duration of Off and On parasol RGCs do not noticeably differ (Jepson et al., 2013). Notably, these differences parallel the findings that black drives macaque parvocellular V1 cortex better than white, and that there is no preference for black in magnocellular V1 (Xing et al., 2010).

### Technical questions

One might ask whether the difference in broadening between intraretinal and extraretinal axons was due to our recording the former *ex vivo* and the latter *in vivo*. In one of three optic nerve axons we were able to record from, spikes broadened in response to the same interspike intervals that broadened spikes in the nerve fiber layer (see Results). Also, spikes resisted broadening in a small fraction of the nerve fiber layer axons we recorded from, as in all of the optic chiasm axons we recorded from. Because we found both spike broadening and resistance to spike broadening in the nerve fiber layer and optic nerve, neither seems attributable to our recording conditions. This is consistent with our recent finding that C spikes slow the conduction velocity of F spikes in compound action potential recordings from both *ex vivo* and *in vivo* rat optic nerves (Partida et al., 2018). We tried to record from more single optic nerve axons, both *in vivo*, in isolated portions of optic nerve (Partida et al., 2018), and in an *ex vivo* retina-attached optic nerve preparation (G. Ogata and A.T. Ishida, unpublished observations) like those developed to study retinal inputs to lateral geniculate nucleus (Mooney et al., 1996) and suprachiasmatic nucleus (Wong et al., 2007). However, none of the electrodes we tested (e.g., glass patch-clamp electrodes, carbon fiber electrodes, various sharp-tipped metal electrodes), including the type we used to record from optic chiasm, allowed us to record from additional optic nerve axons.

One might also ask whether the difference in broadening between intraretinal and extraretinal axons was due to our use of different rat strains for our *ex vivo* and *in vivo* recordings. We think this is unlikely because the spikes in these strains are similar in every other property we assessed. Specifically, we found similar C spike durations (Fig. 9) and interspike intervals (Fig. 4), similar cell type-specific differences (Fig. 9), and activity-induced propagation slowing (Partida et al., 2018), in them. We know of no differences in synaptic inputs (Pitcher et al., 2007), neuromodulator responses (Gulledge and Jaffe, 1998), or spike-generating mechanisms that would favor broadening in one strain and lack of broadening in the other. Moreover, similar RGC receptive field sizes (Heine and Passaglia, 2011) and flash electroretinogram kinetics (Polosa et al., 2016) have been found in control Brown Norway and Long–Evans rats.

Lastly, because spikes did not broaden in the optic tract of rhesus macaques (Fig. 5) ventilated with isoflurane and N_2_O/O_2_ to maintain anesthesia (Sincich et al., 2007), we would not attribute the lack of spike broadening in optic chiasm to our use of ketamine and xylazine to maintain anesthesia during our *in vivo* rat recordings. Instead, the results in Figures 2–5 raise the possibility that lack of spike broadening is a general property of the extraretinal portion of mammalian RGC axons.

## Synthesis

Reviewing Editor: Quentin Pittman, University of Calgary Decisions are customarily a result of the Reviewing Editor and the peer reviewers coming together and discussing their recommendations until a consensus is reached. When revisions are invited, a fact-based synthesis statement explaining their decision and outlining what is needed to prepare a revision will be listed below. The following reviewer(s) agreed to reveal their identity: Wallace Thoreson.

This is a clear and well-written study showing that while interspike frequency and history of light exposure can alter spike duration in fibers as they pass through the retina, these frequency and light-dependent differences in spike duration are lost when axons exit the eye through the optic nerve. There are, however, also differences in spike duration among cell types and these persist in extraretinal axons. Thus, extraretinal spikes from a particular type of ganglion cells have a characteristic and consistent spike duration regardless of spike frequency or the prior history of illumination. In the discussion, the authors consider possible functional consequences (e.g., the possibility that narrow spikes may drive thalamic neurons better than wide spikes). The paper provides a very detailed description of their recording and analytical methodology. the major suggestion is that readers of the paper would benefit from a discussion of some possible mechanisms for the normalization of extra-retinal spikes and also deal with other possible (artefactual) mechanisms arising from the preparations.

1. One potential cause of the abovementioned difference is that the intraretinal axons were recorded ex vivo, whereas the extraretinal axons were recorded in vivo. So the main concern is that perhaps these stimulus-dependent differences in intraretinal axons are an artefact of the preparation. Ideally the authors should generate eyecups that remain attached to the optic nerve (e.g. Wong et al. 2007, J Biol Rhythms 22:400-10), which would enable spike recording from the optic nerve before and during illumination of the retina. If this capability is not within the authors repertoire, this should be addressed in the discussion.

2. The ex vivo and in vivo experiments used different strains of rats. Though it is fairly unlikely that this strain difference contributed to the ex vivo versus in vivo difference, ideally all experiments should have used the same rat strain. Why were different strains used? Again this should be dealt with in the discussion.

3. It is rather surprising that both ON and OFF RGCs’ spikes were broadened upon light presentation, because for the ON cells, illumination increases glutamate receptor activation, but for the OFF cells, illumination reduces glutamate receptor activation. A possibility the authors did not consider is nonspecific photoelectric effects of the stimulus light. Such effects must be ruled out.

4. Though the spike width changes were statistically significant, most of them were very small. Is there any evidence in the published literature that such a small effect size is physiologically relevant? The authors cited a paper saying that small changes in spike broadening can have significant effects on presynaptic release, but both reviewers commented upon the small size of these effects so it would not hurt to have them address this issue in more detail.

5. Regarding possible mechanisms for normalization of extra-retinal spikes, there are at least two considerations. One is that myelination effectively decreases axonal capacitance and thus speeds the membrane time constant. The other is that once action potential threshold is exceed, the rapid regenerative activation provided by the high density of sodium channels clustered in the axon initial segment would tend to make all action potentials equally fast. Does spike normalization in myelinated nerves involve speeding of slower spikes arriving from intraretinal fibers? Or slowing of faster spikes? From the comparison of MEA and tungsten recordings in different cell types, it appears that chiasm spikes may be broader than intraretinal spikes but perhaps this difference is more due to technical differences in the recording method.

Minor:

1) Lines 65-66. The statement that “differences in spike duration of intraretinal axons persist in extraretinal axons” does not seem to agree with the results. Perhaps what the authors are trying to say is that “the differences among cell types in spike duration ...”

2) Line 181. Are these methods in reference to primate recordings? Or do they also apply to rat recordings?

3) Line 200. What is the rationale for choosing d2V/dt2? Is it simply empirical?

4) Line 377. Does “a limited number” mean n=3?

5) The Results and Discussion sections are needlessly long. We recommend tightening up the writing in both.

6) Please list the ingredients of the “Ames medium modified to contain 0.1 mM Ca2+ and 3.4 mM Mg2+”.

7) The effects of dopamine are probably too slow to account for the increase in spike width seen within 100 ms after light onset.

## References

[B1] Baden T, Berens P, Franke K, Román Rosón M, Bethge M, Euler T (2016) The functional diversity of retinal ganglion cells in the mouse. Nature 529:345–350. 10.1038/nature16468 26735013 PMC4724341

[B2] Baker GE, Stryker MP (1990) Retinofugal fibres change conduction velocity and diameter between the optic nerve and tract in ferrets. Nature 344:342–345. 10.1038/344342a0 2314474

[B3] Bean BP (2007) The action potential in mammalian central neurons. Nat Rev Neurosci 8:451–465. 10.1038/nrn2148 17514198

[B4] Bernstein SL, Meister M, Zhuo J, Gullapalli RP (2016) Postnatal growth of the human optic nerve. Eye (Lond) 30:1378–1380. 10.1038/eye.2016.141 27419835 PMC5129860

[B5] Berson DM (2008) Retinal ganglion cell types and their central projections In: The senses: A comprehensive reference (Masland RH, Albright TD, eds), pp 491–520. San Diego: Academic.

[B6] Berson DM, Dunn FA, Takao M (2002) Phototransduction by retinal ganglion cells that set the circadian clock. Science 295:1070–1073. 10.1126/science.1067262 11834835

[B7] Blitz DM, Regehr WG (2003) Retinogeniculate synaptic properties controlling spike number and timing in relay neurons. J Neurophysiol 90:2438–2450. 10.1152/jn.00562.2003 14534270

[B8] Boiko T, Van Wart A, Caldwell JH, Levinson SR, Trimmer JS, Matthews G (2003) Functional specialization of the axon initial segment by isoform-specific sodium channel targeting. J Neurosci 23:2306–2313. 12657689 10.1523/JNEUROSCI.23-06-02306.2003 PMC6742039

[B9] Bowling DB, Michael CR (1980) Projection patterns of single physiologically characterized optic tract fibres in cat. Nature 286:899–902. 10.1038/286899a0 7412871

[B10] Brenowitz SD, Regehr WG (2007) Reliability and heterogeneity of calcium signaling at single presynaptic boutons of cerebellar granule cells. J Neurosci 27:7888–7898. 10.1523/JNEUROSCI.1064-07.2007 17652580 PMC6672738

[B11] Bruesch SR, Arey LB (1942) The number of myelinated and unmyelinated fibers in the optic nerve of vertebrates. J Comp Neurol 77:631–665. 10.1002/cne.900770310

[B12] Burke W, Sefton AJ (1966) Discharge patterns of principal cells and interneurones in lateral geniculate nucleus of rat. J Physiol 187:201–212. 10.1113/jphysiol.1966.sp008083 5972163 PMC1395976

[B13] Cleland BG, Lee BB (1985) A comparison of visual responses of cat lateral geniculate nucleus neurones with those of ganglion cells afferent to them. J Physiol 369:249–268. 10.1113/jphysiol.1985.sp015899 4093882 PMC1192647

[B14] Cleland BG, Levick WR (1974) Brisk and sluggish concentrically organized ganglion cells in the cat’s retina. J Physiol 240:421–456. 10.1113/jphysiol.1974.sp010617 4421622 PMC1331023

[B15] Cleland BG, Dubin MW, Levick WR (1971) Sustained and transient neurones in the cat’s retina and lateral geniculate nucleus. J Physiol 217:473–496. 10.1113/jphysiol.1971.sp009581 5097609 PMC1331787

[B16] Curcio CA, Allen KA (1990) Topography of ganglion cells in human retina. J Comp Neur 300:5–25. 10.1002/cne.903000103 2229487

[B17] Faber ES, Sah P (2003) Ca^2+^-activated K^+^ (BK) channel inactivation contributes to spike broadening during repetitive firing in the rat lateral amygdala. J Physiol 552:483–497. 10.1113/jphysiol.2003.050120 14561831 PMC2343392

[B18] Fleidervish IA, Friedman A, Gutnick MJ (1996) Slow inactivation of Na^+^ current and slow cumulative spike adaptation in mouse and guinea-pig neocortical neurones in slices. J Physiol 493:83–97. 10.1113/jphysiol.1996.sp021366 8735696 PMC1158952

[B19] Foster RE, Connors BW, Waxman SG (1982) Rat optic nerve: Electrophysiological, pharmacological and anatomical studies during development. Brain Res 255:371–386. 10.1016/0165-3806(82)90005-0 7066695

[B20] Foust A, Popovic M, Zecevic D, McCormick DA (2010) Action potentials initiate in the axon initial segment and propagate through axon collaterals reliably in cerebellar Purkinje neurons. J Neurosci 30:6891–6902. 10.1523/JNEUROSCI.0552-10.2010 20484631 PMC2990270

[B21] Freeman DK, Heine WF, Passaglia CL (2008) The maintained discharge of rat retinal ganglion cells. Vis Neurosci 25:535–548. 10.1017/S095252380808067X 18634718 PMC2716752

[B22] Fukada Y (1971) Receptive field organization of cat optic nerve fibers with special reference to conduction velocity. Vision Res 11:209–226. 10.1016/0042-6989(71)90186-6 5579837

[B23] Fukuda Y (1977) A three-group classification of rat retinal ganglion cells: Histological and physiological studies. Brain Res 119:327–334. 10.1016/0006-8993(77)90314-6 830390

[B24] Fukuda Y, Stone J (1974) Retinal distribution and central projections of Y-, X-, and W-cells of the cat’s retina. J Neurophysiol 37:749–772. 10.1152/jn.1974.37.4.749 4837773

[B25] Gaze RM, Peters A (1961) The development, structure and composition of the optic nerve of Xenopus laevis (Daudin). Q J Exp Physiol Cogn Med Sci 46:299–309. 10.1113/expphysiol.1961.sp001548 13897397

[B26] Geiger JR, Jonas P (2000) Dynamic control of presynaptic Ca^2+^ inflow by fast-inactivating K^+^ channels in hippocampal mossy fiber boutons. Neuron 28:927–939. 10.1016/S0896-6273(00)00164-1 11163277

[B27] Giese KP, Storm JF, Reuter D, Fedorov NB, Shao LR, Leicher T, Pongs O, Silva AJ (1998) Reduced K^+^ channel inactivation, spike broadening, and after-hyperpolarization in K_v_beta1.1-deficient mice with impaired learning. Learn Mem 5:257–273. 10454353 PMC311244

[B28] Gollisch T, Meister M (2008) Rapid neural coding in the retina with relative spike latencies. Science 319:1108–1111. 10.1126/science.1149639 18292344

[B29] Granit R (1955) Centrifugal and antidromic effects on ganglion cells of retina. J Neurophysiol 18:388–411. 10.1152/jn.1955.18.4.388 13243145

[B30] Greschner M, Field GD, Li PH, Schiff ML, Gauthier JL, Ahn D, Sher A, Litke AM, Chichilnisky EJ (2014) A polyaxonal amacrine cell population in the primate retina. J Neurosci 34:3597–3606. 10.1523/JNEUROSCI.3359-13.2014 24599459 PMC3942577

[B31] Gulledge AT, Jaffe DB (1998) Dopamine decreases the excitability of layer V pyramidal cells in the rat prefrontal cortex. J Neurosci 18:9139–9151. 10.1523/JNEUROSCI.18-21-09139.1998 9787016 PMC6793538

[B32] Hammer S, Monavarfeshani A, Lemon T, Su J, Fox MA (2015) Multiple retinal axons converge onto relay cells in the adult mouse thalamus. Cell Rep 12:1575–1583. 10.1016/j.celrep.2015.08.003 26321636 PMC5757867

[B33] Hartline K (1938) The response of single optic nerve fibers of the vertebrate eye to illumination of the retina. Am J Physiol 121:400–415. 10.1152/ajplegacy.1938.121.2.400

[B34] Hayashida Y, Ishida AT (2004) Dopamine receptor activation can reduce voltage-gated Na^+^ current by modulating both entry into and recovery from inactivation. J Neurophysiol 92:3134–3141. 10.1152/jn.00526.2004 15486428 PMC3236027

[B35] Hayashida Y, Rodríguez CV, Ogata G, Partida GJ, Oi H, Stradleigh TW, Lee SC, Felipe Colado A, Ishida AT (2009) Inhibition of adult rat retinal ganglion cells by D1-type dopamine receptor activation. J Neurosci 29:15001–15016. 10.1523/JNEUROSCI.3827-09.2009 19940196 PMC3236800

[B36] Heine WF, Passaglia CL (2011) Spatial receptive field properties of rat retinal ganglion cells. Vis Neurosci 28:403–417. 10.1017/S0952523811000307 21944166 PMC5130229

[B37] Hidaka S, Ishida AT (1998) Voltage-gated Na^+^ current availability after step- and spike-shaped conditioning depolarizations of retinal ganglion cells. Pflügers Arch 436:497–508. 10.1007/s004240050664 9683721

[B38] Hildebrand C, Remahl S, Waxman SG (1985) Axo-glial relations in the retina-optic nerve junction of the adult rat: Electron-microscopic observations. J Neurocytol 14:597–617. 10.1007/bf01200800 4067610

[B39] Hokoç JN, Oswaldo-Cruz EO (1978) Quantitative analysis of the opossum’s optic nerve: An electron microscope study. J Comp Neurol 178:773–782. 10.1002/cne.901780411 632381

[B40] Holmberg K (1972) Fine structure of the optic tract in the Atlantic hagfish, Myxine glutinosa. Acta Zool 53:165–171. 10.1111/j.1463-6395.1972.tb00584.x

[B41] Hughes TE, Carey RG, Vitorica J, de Blas AL, Karten HJ (1989) Immunohistochemical localization of GABA_A_ receptors in the retina of the new world primate Saimiri sciureus. Vis Neurosci 2:565–581. 10.1017/s0952523800003503 2562111

[B42] Jackson MB, Konnerth A, Augustine GJ (1991) Action potential broadening and frequency-dependent facilitation of calcium signals in pituitary nerve terminals. Proc Natl Acad Sci USA 88:380–384. 10.1073/pnas.88.2.380 1988937 PMC50814

[B43] Jepson LH, Hottowy P, Mathieson K, Gunning DE, Dabrowski W, Litke AM, Chichilnisky EJ (2013) Focal electrical stimulation of major ganglion cell types in the primate retina for the design of visual prostheses. J Neurosci 33:7194–7205. 10.1523/JNEUROSCI.4967-12.2013 23616529 PMC3735130

[B44] Juusola M, Robinson HP, de Polavieja GG (2007) Coding with spike shapes and graded potentials in cortical networks. Bioessays 29:178–187. 10.1002/bies.20532 17226812

[B45] Khaliq ZM, Raman IM (2006) Relative contributions of axonal and somatic Na channels to action potential initiation in cerebellar Purkinje neurons. J Neurosci 26:1935–1944. 10.1523/JNEUROSCI.4664-05.2006 16481425 PMC6674931

[B46] Kim KJ, Rieke F (2003) Slow Na^+^ inactivation and variance adaptation in salamander retinal ganglion cells. J Neurosci 23:1506–1516. 12598639 10.1523/JNEUROSCI.23-04-01506.2003 PMC6742238

[B47] Kirk DL, Cleland BG, Levick WR (1975) Axonal conduction latencies of cat retinal ganglion cells. J Neurophysiol 38:1395–1402. 10.1152/jn.1975.38.6.1395 1221078

[B48] Kolb H, Cuenca N, Wang HH, Dekorver L (1990) The synaptic organization of the dopaminergic amacrine cell in the cat retina. J Neurocytol 19:343–366. 10.1007/bf01188404 2391538

[B49] Kole MH, Letzkus JJ, Stuart GJ (2007) Axon initial segment Kv1 channels control axonal action potential waveform and synaptic efficacy. Neuron 55:633–647. 10.1016/j.neuron.2007.07.031 17698015

[B50] Kusano K, Livengood DR, Werman R (1967) Tetraethylammonium ions: Effect of presynaptic injection on synaptic transmission. Science 155:1257–1259. 10.1126/science.155.3767.1257 6018645

[B51] Lang J, Reiter W (1985) Mass of the optic nerve, the optic chiasm and the optic tract of practical medical importance. Gegenbaurs Morphol Jahrb 131:777–795. 4085754

[B52] Li BY, Glazebrook P, Kunze DL, Schild JH (2011) K_Ca_1.1 channel contributes to cell excitability in unmyelinated but not myelinated rat vagal afferents. Am J Physiol Cell Physiol 300:C1393–C1403. 10.1152/ajpcell.00278.2010 21325638 PMC3118615

[B53] Li PH, Gauthier JL, Schiff M, Sher A, Ahn D, Field GD, Greschner M, Callaway EM, Litke AM, Chichilnisky EJ (2015) Anatomical identification of extracellularly recorded cells in large-scale multielectrode recordings. J Neurosci 35:4663–4675. 10.1523/JNEUROSCI.3675-14.2015 25788683 PMC4363392

[B54] Lipton SA, Tauck DL (1987) Voltage-dependent conductances of solitary ganglion cells dissociated from the rat retina. J Physiol 385:361–391. 10.1113/jphysiol.1987.sp016497 2443669 PMC1192350

[B55] Masland RH, Ames A 3rd (1976) Responses to acetylcholine of ganglion cells in an isolated mammalian retina. J Neurophysiol 39:1220–1235. 10.1152/jn.1976.39.6.1220 993829

[B56] Mastronarde DN (1987) Two classes of single-input X-cells in cat lateral geniculate nucleus. I. Receptive-field properties and classification of cells. J Neurophysiol 57:357–380. 10.1152/jn.1987.57.2.357 3559684

[B57] Matheson DF (1970) Some quantitative aspects of myelination of the optic nerve in rat. Brain Res 24:257–269. 10.1016/0006-8993(70)90105-8 5490286

[B58] McIlwain JT (1964) Receptive fields of optic tract axons and lateral geniculate cells: Peripheral extent and barbiturate sensitivity. J Neurophysiol 27:1154–1173. 10.1152/jn.1964.27.6.1154 14223976

[B59] Meeks JP, Jiang X, Mennerick S (2005) Action potential fidelity during normal and epileptiform activity in paired soma-axon recordings from rat hippocampus. J Physiol 566:425–441. 10.1113/jphysiol.2005.089086 15890699 PMC1464751

[B60] Meister M, Pine J, Baylor DA (1994) Multi-neuronal signals from the retina: Acquisition and analysis. J Neurosci Methods 51:95–106. 10.1016/0165-0270(94)90030-2 8189755

[B61] Milner ES, Do MTH (2017) A population representation of absolute light intensity in the mammalian retina. Cell 171:865–876. 10.1016/j.cell.2017.09.005 28965762 PMC6647834

[B62] Moody GJ, Oke RB, Thomas JDR (1969) The influence of light on silver-silver chloride electrodes. Analyst 94:803–804. 10.1039/an9699400803

[B63] Mooney R, Penn AA, Gallego R, Shatz CJ (1996) Thalamic relay of spontaneous retinal activity prior to vision. Neuron 17:P863–P874. 10.1016/S0896-6273(00)80218-4 8938119

[B64] Morgan JL, Berger DR, Wetzel AW, Lichtman JW (2016) The fuzzy logic of network connectivity in mouse visual thalamus. Cell 165:192–206. 10.1016/j.cell.2016.02.033 27015312 PMC4808248

[B65] Morin LP, Studholme KM (2014) Retinofugal projections in the mouse. J Comp Neurol 522:3733–3753. 10.1002/cne.23635 24889098 PMC4142087

[B66] Mosinger JL, Yazulla S, Studholme KM (1986) GABA-like immunoreactivity in the vertebrate retina: A species comparison. Exp Eye Res 42:631–644. 10.1016/0014-4835(86)90052-7 3487464

[B67] O’Brien BJ, Isayama T, Richardson R, Berson DM (2002) Intrinsic physiological properties of cat retinal ganglion cells. J Physiol 538:787–802. 10.1113/jphysiol.2001.013009 11826165 PMC2290089

[B68] Partida GJ, Fasoli A, Fogli Iseppe A, Ogata G, Johnson JS, Thambiaiyah V, Passaglia CL, Ishida AT (2018) Autophosphorylated CaMKII facilitates spike propagation in rat optic nerve. J Neurosci 38:8087–8105. 10.1523/JNEUROSCI.0078-18.2018 30076212 PMC6136153

[B69] Pitcher TL, Wickens JR, Reynolds JN (2007) Differences in striatal spiny neuron action potentials between the spontaneously hypertensive and Wistar-Kyoto rat strains. Neuroscience 146:135–142. 10.1016/j.neuroscience.2007.01.003 17320302

[B70] Polosa A, Bessaklia H, Lachapelle P (2016) Strain differences in light-induced retinopathy. PLoS One 11:e0158082. 10.1371/journal.pone.0158082 27355622 PMC4927188

[B71] Popovic MA, Foust AJ, McCormick DA, Zecevic D (2011) The spatio-temporal characteristics of action potential initiation in layer 5 pyramidal neurons: A voltage imaging study. J Physiol 589:4167–4187. 10.1113/jphysiol.2011.209015 21669974 PMC3180577

[B72] Ramón y Cajal S (1972) The structure of the retina. Springfield, IL: CC Thomas.

[B73] Reid RC, Victor JD, Shapley RM (1997) The use of m-sequences in the analysis of visual neurons: Linear receptive field properties. Vis Neurosci 14:1015–1027. 10.1017/S0952523800011743 9447685

[B74] Ritzau-Jost A, Delvendahl I, Rings A, Byczkowicz N, Harada H, Shigemoto R, Hirrlinger J, Eilers J, Hallermann S (2014) Ultrafast action potentials mediate kilohertz signaling at a central synapse. Neuron 84:152–163. 10.1016/j.neuron.2014.08.036 25220814

[B75] Rowan MJ, DelCanto G, Yu JJ, Kamasawa N, Christie JM (2016) Synapse-level determination of action potential duration by K^+^ channel clustering in axons. Neuron 91:370–383. 10.1016/j.neuron.2016.05.035 27346528 PMC4969170

[B76] Sabatini BL, Regehr WG (1997) Control of neurotransmitter release by presynaptic waveform at the granule cell to Purkinje cell synapse. J Neurosci 17:3425–3435. 9133368 10.1523/JNEUROSCI.17-10-03425.1997 PMC6573699

[B77] Sabatini BL, Regehr WG (1999) Timing of synaptic transmission. Annu Rev Physiol 61:521–542. 10.1146/annurev.physiol.61.1.521 10099700

[B78] Sakmann B, Creutzfeldt OD (1969) Scotopic and mesopic light adaptation in the cat’s retina. Pflügers Arch 313:168–185. 10.1007/bf00586245 5390975

[B79] Salami M, Itami C, Tsumoto T, Kimura F (2003) Change of conduction velocity by regional myelination yields constant latency irrespective of distance between thalamus and cortex. Proc Natl Acad Sci USA 100:6174–6179. 10.1073/pnas.0937380100 12719546 PMC156345

[B80] Sekirnjak C, Hottowy P, Sher A, Dabrowski W, Litke AM, Chichilnisky EJ (2006) Electrical stimulation of mammalian retinal ganglion cells with multielectrode arrays. J Neurophysiol 95:3311–3327. 10.1152/jn.01168.2005 16436479

[B81] Sekirnjak C, Hottowy P, Sher A, Dabrowski W, Litke AM, Chichilnisky EJ (2008) High-resolution electrical stimulation of primate retina for epiretinal implant design. J Neurosci 28:4446–4456. 10.1523/JNEUROSCI.5138-07.2008 18434523 PMC2681084

[B82] Sincich LC, Adams DL, Economides JR, Horton JC (2007) Transmission of spike trains at the retinogeniculate synapse. J Neurosci 27:2683–2692. 10.1523/JNEUROSCI.5077-06.2007 17344406 PMC6672514

[B83] Singer W, Pöppel E, Creutzfeldt O (1972) Inhibitory interaction in the cat’s lateral geniculate nucleus. Exp Brain Res 14:210–226. 10.1007/bf00234800 5016590

[B84] Stanford LR (1987) Conduction velocity variations minimize conduction time differences among retinal ganglion cell axons. Science 238:358–360. 10.1126/science.3659918 3659918

[B85] Stone J, Freeman RB Jr (1971) Conduction velocity groups in the cat’s optic nerve classified according to their retinal origin. Exp Brain Res 13:489–497. 10.1007/bf00234279 5137298

[B86] Stone J, Makarov F, Holländer H (1995) The glial ensheathment of the soma and axon hillock of retinal ganglion cells. Vis Neurosci 12:273–279. 10.1017/s0952523800007951 7786848

[B87] Straatsma BR, Heckenlively JR, Foos RY, Shahinian JK (1979) Myelinated retinal nerve fibers associated with ipsilateral myopia, amblyopia, and strabismus. Am J Ophthalmol 88:506–510. 10.1016/0002-9394(79)90655-x 484678

[B88] Sumitomo I, Ide K, Iwama K, Arikuni T (1969) Conduction velocity of optic nerve fibers innervating lateral geniculate body and superior colliculus in the rat. Exp Neurol 25:378–392. 10.1016/0014-4886(69)90132-0 4310870

[B89] Terzuolo CA, Araki T (1961) An analysis of intra- versus extracellular potential changes associated with activity of single spinal motoneurons. Ann N Y Acad Sci 94:547–558. 10.1111/j.1749-6632.1961.tb35558.x 13776058

[B90] Tomassy GS, Berger DR, Chen HH, Kasthuri N, Hayworth KJ, Vercelli A, Seung HS, Lichtman JW, Arlotta P (2014) Distinct profiles of myelin distribution along single axons of pyramidal neurons in the neocortex. Science 344:319–324. 10.1126/science.1249766 24744380 PMC4122120

[B91] Ulrich J, Groebke-Lorenz W (1983) The optic nerve in multiple sclerosis: A morphological study with retrospective clinic-pathological correlations. Neuro-Ophthalmol 3:149–159. 10.3109/01658108309009732

[B92] Usrey WM, Reppas JB, Reid RC (1998) Paired-spike interactions and synaptic efficacy of retinal inputs to the thalamus. Nature 395:384–387. 10.1038/26487 9759728

[B93] Utzschneider DA, Thio C, Sontheimer H, Ritchie JM, Waxman SG, Kocsis JD (1993) Action potential conduction and sodium channel content in the optic nerve of the myelin-deficient rat. Proc Biol Sci 254B:245–250. 10.1098/rspb.1993.0153 8108457

[B94] Vaney DI, Levick WR, Thibos LN (1981) Rabbit retinal ganglion cells. Receptive field classification and axonal conduction properties. Exp Brain Res 44:27–33. 10.1007/bf00238746 6168481

[B95] Vigneswaran G, Kraskov A, Lemon RN (2011) Large identified pyramidal cells in macaque motor and premotor cortex exhibit “thin spikes”: Implications for cell type classification. J Neurosci 31:14235–14242. 10.1523/JNEUROSCI.3142-11.2011 21976508 PMC3199219

[B96] Weick M, Demb JB (2011) Delayed-rectifier K channels contribute to contrast adaptation in mammalian retinal ganglion cells. Neuron 71:166–179. 10.1016/j.neuron.2011.04.033 21745646 PMC3134798

[B97] Weyand TG (2007) Retinogeniculate transmission in wakefulness. J Neurophysiol 98:769–785. 10.1152/jn.00929.2006 17553944

[B98] Williams SR, Stuart GJ (1999) Mechanisms and consequences of action potential burst firing in rat neocortical pyramidal neurons. J Physiol 521:467–482. 10.1111/j.1469-7793.1999.00467.x 10581316 PMC2269673

[B99] Wollner DA, Catterall WA (1986) Localization of sodium channels in axon hillocks and initial segments of retinal ganglion cells. Proc Natl Acad Sci USA 83:8424–8428. 10.1073/pnas.83.21.8424 2430289 PMC386941

[B100] Wong KY, Graham DM, Berson DM (2007) The retina-attached SCN slice preparation: An in vitro mammalian circadian visual system. J Biol Rhythms 22:400–410. 10.1177/0748730407305376 17876061

[B101] Wong RCS, Cloherty SL, Ibbotson MR, O’Brien BJ (2012) Intrinsic physiological properties of rat retinal ganglion cells with a comparative analysis. J Neurophysiol 108:2008–2023. 10.1152/jn.01091.2011 22786958

[B102] Xing D, Yeh CI, Shapley RM (2010) Generation of black-dominant responses in V1 cortex. J Neurosci 30:13504–13512. 10.1523/JNEUROSCI.2473-10.2010 20926676 PMC3842489

[B103] Zeck G, Lambacher A, Fromherz P (2011) Axonal transmission in the retina introduces a small dispersion of relative timing in the ganglion cell population response. PLoS One 6:e20810. 10.1371/journal.pone.0020810 21674067 PMC3107248

